# Robot-Aided Magnetic Navigation System for Wireless Capsule Manipulation

**DOI:** 10.3390/mi14020269

**Published:** 2023-01-20

**Authors:** Seyeong Im, Sungjun Kim, Joongho Yun, Jaekwang Nam

**Affiliations:** Department of Robotics, Kwangwoon University, Seoul 01897, Republic of Korea

**Keywords:** magnetic capsule, magnetic navigation system, robotic arm, wireless manipulation

## Abstract

Magnetic navigation systems (MNSs) have been developed to use in the diagnosis of gastrointestinal problems. However, most conventional magnetic navigation systems are expensive and have structural problems because of their large weights and volumes. Therefore, this paper proposes C-Mag, a novel compact MNS composed of two electromagnets and a robotic arm. The two electromagnets generate a planar magnetic field, and the robotic arm rotates and translates the electromagnets to manipulate the magnetic capsule in a large 3-dimensional (3-D) space. The C-Mag design considers the payload of the robotic arm and the capacity of the power supply unit. Under these limited conditions, the C-Mag was optimized to generate the maximum magnetic field considering several major factors. Finally, the C-Mag was constructed, and the maximum magnetic field that could be generated in one direction was 18.65 mT in the downward direction. Additionally, the maximum rotating magnetic field was 13.21 mT, which was used to manipulate the capsule. The performance was verified by measuring the generated magnetic field, and it matched well with the simulated result. Additionally, the path-following experiment of the magnetic capsule showed that the proposed C-Mag can effectively manipulate the magnetic capsule in 3-D space using the robotic arm. This study is expected to contribute to the further development of magnetic navigation systems to treat gastrointestinal problems.

## 1. Introduction

Flexible endoscopy is the standard procedure for diagnosing and treating various gastrointestinal problems. This traditional method is reliable and minimally invasive; however it causes discomfort to the patient because a long flexible cable is pushed into the body through the throat [1]. Wireless capsule endoscopy is an alternative to flexible endoscopy [1,2,3,4,5]. This method uses a pill-sized capsule. The capsule is swallowed, which then passes naturally through the digestive tract, where it captures images for approximately 8 h. This method is considered safe does not cause significant discomfort to the patient [1,2]. However, the developed capsule has several disadvantages because it only passively moves via gastrointestinal peristalsis. One disadvantage is that the capsule needs at least a day to be excreted from the body although the patient does not need to stay in the hospital. In addition, it can be lodged or stuck in a narrow area or a stricture. Furthermore, capsules that move through peristalsis during digestion cannot control the position or posture of the capsules to observe a certain area for diagnostic or therapeutic purposes. This can lead to an unreliable diagnosis for patients [4,5].

To overcome these problems, wireless capsules that can actively move in the gastrointestinal tract, have been studied [1,2,3,4,5,6,7,8]. In particular, magnetic capsules, which are remotely controlled via an external magnetic field to actively move, adjust its posture, and are used to perform various tasks such as sampling, drug delivery, and biopsy [4,5,6,7,8,9,10,11,12]. The external magnetic field is generated by the magnetic navigation system (MNS), and various MNSs have been developed for various purposes [13,14,15,16,17,18,19,20,21,22,23]. Several researchers have investigated MNSs composed of coils, which generates a uniform magnetic field or field gradient [13,14,15,16,17]. However, it must have a large volume to accommodate the human body inside the MNS. Magnetic cores can be used to amplify the magnetic field, thus reducing the volume of the MNS [18,19,20]. In particular, these MNSs can be integrated with a robotic arm to manipulate the capsule in a three-dimensional (3-D) space with a minimum number of electromagnets or permanent magnet which effectively reduce the volume of the MNS. Sikorski et al. developed the robot-aided MNS with a single electromagnet which guides a magnetic catheter [21], but its structure is not appropriate to generate rotating a magnetic field which is used for the rolling motion of the capsule. In contrast, Mahoney et al. developed the robot-aided MNS with a single permanent magnet that can generate a rolling motion of the capsule [22]. However, the MNS based on permanent magnets cannot precisely generate and control a magnetic field compared to the MNS based on electromagnets. Lucarini et al. proposed the robot-aided MNS with two electromagnets which can generate the rolling motion of the capsule [23], but its design did not consider the electrical properties and shape of the electromagnets, such as the shape of the core tip, thus significantly affecting the magnetic field. In addition, the locomotion of the capsule was not verified with a robotic arm.

This study proposes C-Mag, a novel robot-aided MNS with two electromagnets. The proposed C-Mag only uses two electromagnets which generate the planar magnetic field in a small local plane, but the local plane can be translated and rotated by a selective compliance assembly robot arm (SCARA). Thus, relatively small C-Mag can generate an arbitrary magnetic field in a large 3-D space for the capsule. Additionally, the closed magnetic circuit, which prevents magnetic flux leakage, was introduced to maximize the magnetic field of the electromagnets. The C-Mag is optimized considering several major factors such as electrical properties, the output of the power supply unit, and the weight capacity of the SCARA. Subsequently, the performance of the constructed C-Mag is verified using a prototype magnetic capsule.

## 2. Development of the C-Mag

### 2.1. Magnetic Torque and Force of the C-Mag

A magnetic robot under an external magnetic field experiences magnetic torque and force that can be expressed as follows:

(1) $$\vec{F}=\left(\vec{m}\cdot \nabla \right)\vec{B}$$

(2) $$\vec{T}=\left(\vec{m}\times \vec{B}\right)$$

where $\vec{m}$ is the magnetic moment of the robot and $\vec{B}$ is the external magnetic field. The torque and force are used to generate the rolling and translation motions of the capsule, respectively. If the actuation system is composed of more than four electromagnets, the capsule can generate both rolling and translation motions in a 2-D plane. However, such an actuation system would be more complex and heavier than a system using two electromagnets. As 2-D translation motion is not essential for the capsule to reach a target area, we constructed the C-Mag with two electromagnets and only utilized the magnetic torque to rotate the capsule. The capsule could then be moved via rolling motion. Since both electromagnets were placed only on the upper side of the capsule, the electromagnets attracted the capsule during operation. However, it is assumed that the capsule is sufficiently heavy to maintain a contact with the ground during the rotating motion. Additionally, this 2-D planar rolling motion can be extended to a 3-D rolling motion by combining the electromagnets with SCARA.

### 2.2. Structure of the C-Mag

**Figure 1:**
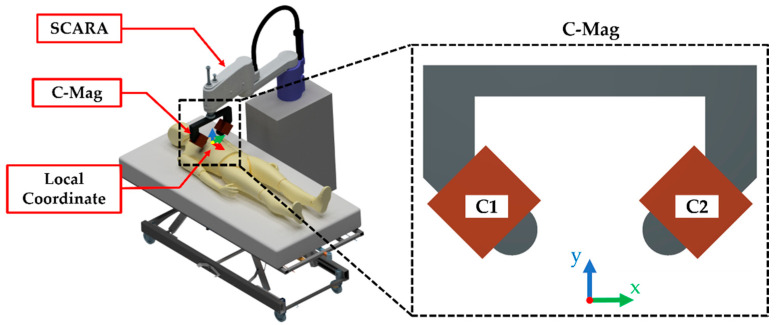
Concept of the C-Mag with the SCARA to generate 3-D rolling motion of a magnetic capsule.

**Figure 2:**
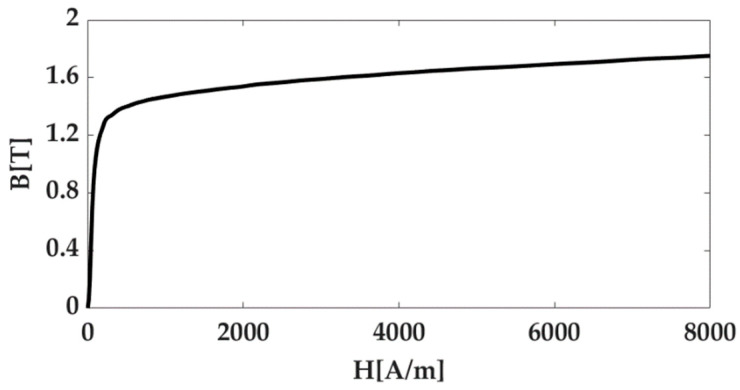
BH curve of the steel sheet (30PNF1600) for the C-Mag. These data are provided courtesy of POSCO.

Figure 1 shows the proposed C-Mag combined with SCARA. The C-Mag has a local coordinate system, and a 2-D rotating magnetic field can be generated in the local *yz*-plane while the local plane can be translated and rotated by the SCARA. The rotation about the global *z*-axis enables the C-Mag to generate a 3-D rotating magnetic field, and the translation in the global *xy*-plane enables the C-Mag to accommodate a relatively large human body. The C-Mag uses two magnetic cores to amplify the magnetic field generated by the coils. However, these cores produce large iron losses when using a time-varying magnetic field. Thus, laminated cores are introduced to prevent the iron loss in C-Mag. In this study, we use a steel sheet (30PNF1600) with a thickness of 0.3 mm for the lamination, and its magnetic flux density is 1.64 T at the saturation point, as shown in Figure 2. As the core is laminated using the same steel sheets, it core has a rectangular cross-section. The thickness of the laminated core is determined to be 43.8 mm, considering the mechanical joint of SCARA.

**Figure 3:**
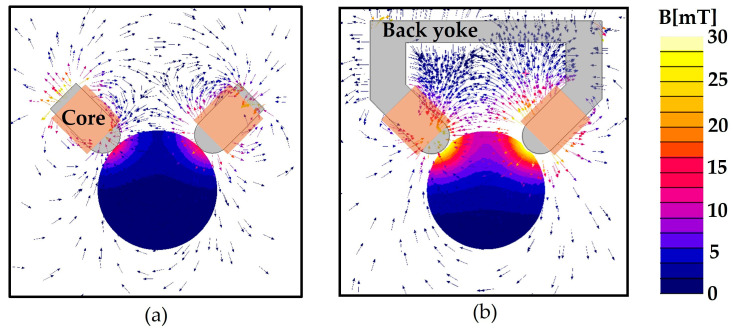
Magnetic flux density distribution around electromagnets. (**a**) Two electromagnets with the core; (**b**) Two electromagnets with the core and back yoke.

Although the magnetic field is amplified by the magnetic cores, the amplified magnetic field can be leaked from back of the electromagnets. To minimize the magnetic leakage, a closed magnetic circuit is constructed by connecting the two cores with a back yoke. The C-Mag can then generate a larger magnetic field with the same output power as the other case without the back yoke. To verify this concept, the performances of the C-Mag with and without the back yoke is compared, as shown in Figure 3. The 3-D magnetic finite element (FE) method is used for the simulation. Each set of electromagnets has the same number of turns of wire (1131 turns) and applied current (1 A). Figure 3 shows that the magnetic field generated by the electromagnets with the back yoke effectively passes through the back yoke with a small magnetic leakage, and the magnetic field is focused on the workspace between the cores. Consequently, the magnetic field at the center point doubles in that of the electromagnets with the back yoke.

### 2.3. Effect of the Core Tip

**Figure 4:**
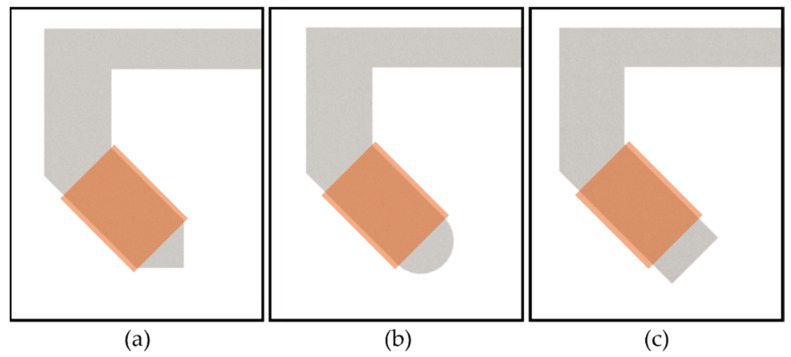
Various core tips for the C-Mag. (**a**) Arrow tip. (**b**) Round tip. (**c**) Square tip.

The shape of the core tip is an important parts of the C-Mag because the distribution of the magnetic field in the workspace depends on it. Three core tips are considered for the C-Mag, as shown in Figure 4. For each case, the simulation was conducted with the same number of turns of wire (1131 turns) and an applied current of 1 A; the magnetic field at the center point was then calculated. The calculated magnetic fields were 1.51 mT, 1.75 mT, and 1.96 mT. The strongest magnetic field was generated with the square tip because it had the smallest reluctance owing to the wide and short magnetic path between the cores. However, as the applied current gradually increased, the sharp edges of the square tip became easily saturated. As the nonlinear property of saturated iron makes it difficult to control the C-Mag, saturation is not desired. Thus, a round tip without an edge is considered a possible option. If the maximum current of 15 A is applied to the round and square tips, both tips generate a similar magnetic field of 22.6 mT and 23.5 mT, respectively. However, if we consider the currents that do not saturate the core, the available maximum currents are calculated as 9.87 A and 4.41 A for round and square tips, and both tips generate a magnetic field of 18.65 mT and 9.84 mT, respectively. Thus, a round tip was selected for the C-Mag considering the magnetic saturation effect.

### 2.4. Effect of the Tilted Angle of the Core

**Figure 5:**
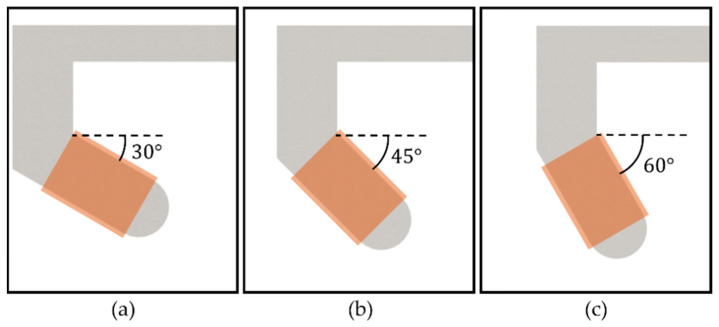
Various tilted angles of the core for the C-Mag. (**a**) 30°. (**b**) 45°. (**c**) 60°.

The effect of the tilted angle of the core on the magnetic field was analyzed. Three FE models with the tilted angles of 30°, 45°, and 60° are considered, as shown in Figure 5. Each simulation was performed with the same number of turns of wire (1131 turns) and applied current (1 A), and the magnetic fields at the center point are calculated when the distance between cores (*d*_g_) was maintained at 20 cm. The calculated magnetic fields were 1.70 mT, 1.75 mT, and 1.54 mT. It can beobserved the first two values were not significantly different. However, the core with the tilted angle of 30° has the greatest weight, whereas the total weight of the core and coil is limited by the maximum payload of the SCARA. Thus, the volume of the coil cannot be significantly increased during optimization. Thus, the core with 45°, which generates the strongest magnetic field, was selected as the tilted angle of the core.

### 2.5. Optimal Design of the C-Mag to Maximize the Generated Magnetic Field

**Figure 6:**
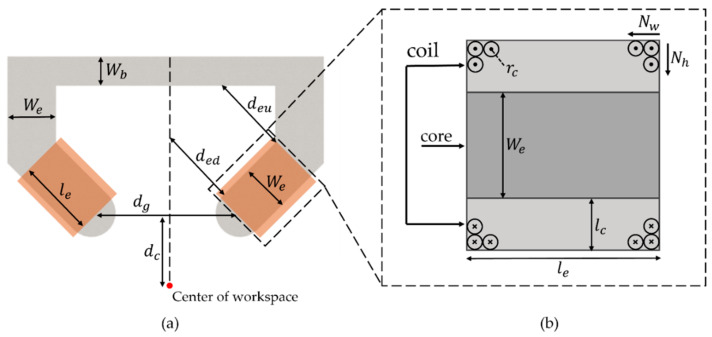
Design variables of the C-Mag: (**a**) design variables of the magnetic core; and (**b**) design variables of the coil.

**Figure 7:**
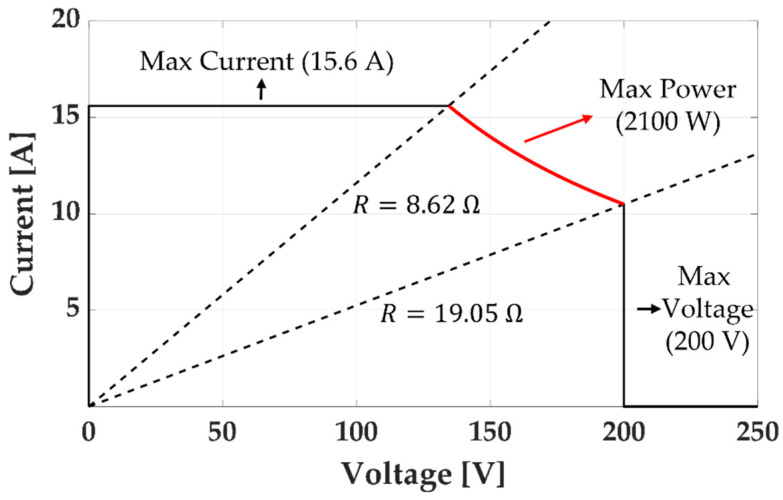
Output range of the power supply unit (3001iX by California Instruments).

We conducted a parametric study of the C-Mag to generate the maximum magnetic field at the center of the workspace. Figure 6 shows the design variables of the C-Mag, and an FE model was used for the simulation. First, we changed the design variables of the core, and the design variables of the coil were calculated to satisfy the weight and resistance constraints. The weight constraint was that the C-Mag must not exceed 20 kg because of the maximum payload of the SCARA. The resistance constraint was that the resistance of the coil must be in the range of 8.62 Ω ≤ $R_{k}$ ≤ 19.05 Ω to utilize the maximum power of the power supply unit (3001iX by California Instruments) as shown in Figure 7. As the resistance can be increased during operation, a minimum resistance of 8.62 Ω was used for the coil.

During the parametric study, the distance between the cores ($d_{g}$) was fixed at 20 cm to maintain the same workspace. Additionally, the center of the workspace from the core ($d_{c}$) was supposed to be 10 cm, considering the size of the human abdomen. The width of the back yoke ($w_{b}$) is not an important design variable because it hardly affects the magnetic field in the workspace unless it is saturated. Thus, $w_{b}$ was fixed at 4 cm, which is sufficiently wide to prevent saturation of the back yoke. To equalize the space where the coils should be wound, the same distance was used for $d_{ed}$ and $d_{eu}$. In contrast, the width and length of the electromagnet ($w_{e}$ and $l_{e}$) significantly affected the magnetic field in the workspace; therefore, we simulated the magnetic field at the center of the workspace for different $w_{e}$ and $l_{e}$ when the maximum power of the power supply unit was applied.

(3) $$\begin{array}{cc} l_{coil} & =2N_{w}\overset{k=N_{h}}{\underset{K=1}{\sum }}\left\{W_{e}+W_{c}+2r_{c}+4r_{c}\left(k-1\right)\right\}\\ & =2N_{w}N_{h}\left(W_{e}+W_{c}+2N_{h}r_{c}\right) \end{array}$$

where $l_{coil}$, and $W_{c}$ are the length of the coil, and thickness of the laminated core, respectively; $N_{w}\;\mathrm{and}\;N_{h}$ are the number of turns of the coil along the axial and radial directions of the electromagnet, respectively. In Equation (3), $N_{w}$ and $N_{h}$ can be rewritten using $r_{c}$ as follows:

(4) $$N_{w}=\frac{l_{e}}{2r_{c}}$$

(5) $$N_{h}=\frac{l_{c}}{2r_{c}}$$

Additionally, the weight of the coil can be calculated using Equations (4) and (5) as follows:

(6) $$M_{cc}=M_{e}+M_{c}$$

where $M_{cc}$, $M_{e}$, and $M_{c}$ denote the total weights of the electromagnet, core, and coil, respectively. We set $M_{cc}$ to 18 kg considering a small margin of the maximum payload of the SCARA (20 kg) because the weight of the mount that connects SCARA and C-mag, is 2 kg. $M_{e}$ is the product of the volume of the core and the density of the steel sheet. $M_{c}$ is an equation consisting of the density of the coil, $r_{c}$, and $l_{coil}.$ The resistance of the coil can be calculated as follows:

(7) $$R_{m}=2\pi r_{c}l_{coil}$$

where $R_{m}$ is the resistance of the coil that utilizes the maximum power of the power supply unit. The resistance of $R_{m}$ was set to 9 Ω. Then, the maximum magnetomotive force for each electromagnet was then calculated by changing the two variables of $w_{e}$ and $l_{e}$ in Equations (6) and (7).

**Figure 8:**
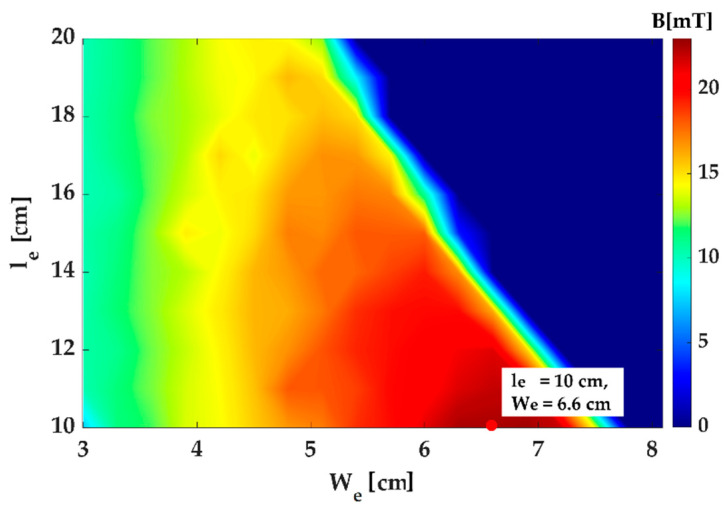
Magnetic field at the center of the workspace for various combinations of $w_{e}$ and $l_{e}$.

A simulation was performed using the calculated magnetomotive force. The results are shown in Figure 8. The optimal point that generates the strongest magnetic field can be observed in the figure. As the larger $w_{e}$ is advantageous for generation a strong magnetic field over the broad area of the workspace, the combination ($w_{e}$ of 6.6 cm and $l_{e}$ of 10 cm) with the maximum $w_{e}$ was selected for the optimal design.

### 2.6. Magnetic Field Generation of the C-Mag

At a given point in the workspace (P), the magnetic field generated by *k*th electromagnet can be expressed as

(8) $$B_{k}\left(P\right)={\tilde{B}}_{k}\left(P\right)i_{k}$$

where ${\tilde{B}}_{k}\left(P\right)$ is the magnetic field of the *k*th electromagnet by the unit current and $i_{k}$ is the current of the *k*th electromagnet. Then, the total magnetic field generated by the two electromagnets can be superposed as

(9) $$B\left(P\right)=\left[\begin{array}{cc} {\tilde{B}}_{1}\left(P\right) & {\tilde{B}}_{2}\left(P\right) \end{array}\right]\left[\begin{array}{c} i_{1}\\ i_{2} \end{array}\right].$$

We assumed that a magnetic robot is located at the center of the workspace ($P_{0}$) during the experiments because it can track the magnetic robot using various localization techniques [24,25,26,27]. Subsequently, the magnetic field generated by C-Mag ${\tilde{B}}_{k}\left(P_{0}\right)$ becomes a constant vector at the given $P_{0}$, and each vector is calculated using FE method [20]. Above the saturation point, nonlinear magnetic properties should be applied in real-time, but this is not considered in this paper.

## 3. Experiments

### 3.1. Construction and Verification of the C-Mag

**Figure 9:**
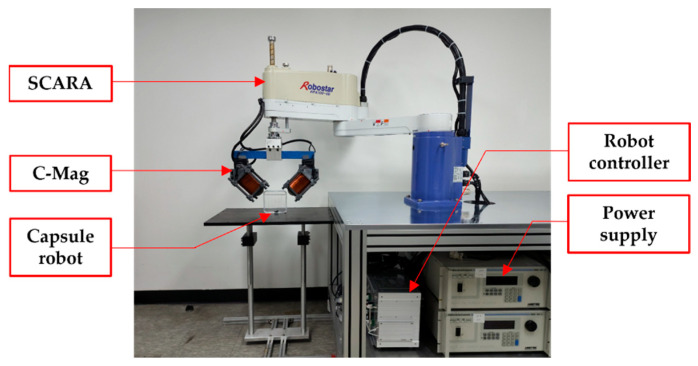
Constructed C-mag and experimental setup.

Figure 9 shows the constructed C-Mag and overall experimental setup. The C-Mag was laminated by the steel sheet (30PNF1600), and its design variable of $w_{e}$, $l_{e}$ were optimized as 6.6 and 10 cm, respectively. The radius ($r_{c}$) of the copper coil was 0.4 mm and it was wound 1131 times to obtain 9 Ω. However, owing various design errors, the measured resistances of the coils of the first electromagnet (C1 in Figure 1) and second electromagnet (C2 in Figure 1) were 10.14 and 9.97 Ω, respectively. The constructed C-Mag has the maximum magnetic field of 18.65 mT that can be generated in one direction in the downward direction and has the maximum rotating magnetic field of 13.21 mT, which is used to manipulate the capsule.

**Figure 10:**
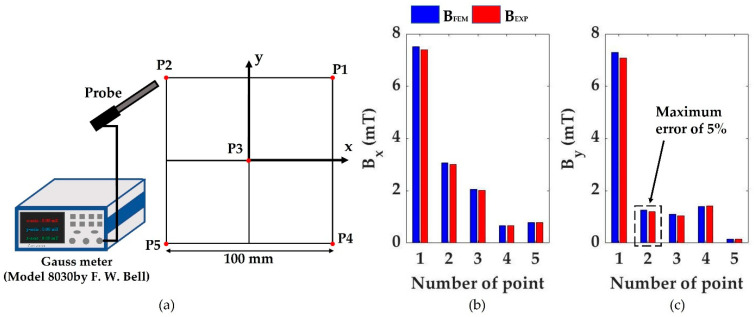
(**a**) Five experimental points in workspace. (**b**) Comparison of magnetic fields in the *x*-direction. (**c**) Comparison of magnetic fields in the *y*-direction.

Subsequently, we verified the constructed C-mag. The difference between the simulation results and the experimental values are compared at five points in the workspace, as shown in Figure 10a. Each magnetic field was measured using a Gaussian probe (Model 8030 by F. W. Bell). Figure 10b shows the comparison between the simulated results and experimental values when a current of 1A is applied to the first electromagnet (C1). This result shows that the magnetic fields generated by the constructed C-Mag fit well with the simulated values with an error of less than 5%. The operation time of the C-Mag was also verified. Each coil of the C-Mag was made of an F-class wire, which has an allowable temperature of 155 °C. If the C-Mag is operated under the applied current of 5.6 A, the C-Mag has an unlimited operation time without overheating, and this current is strong enough to actuate the capsule in a normal environment. In contrast, if the capsule utilizes the maximum current of 9.8 A, at which the core is not saturated, the coil overheats in 78 s. Thus, the maximum current should be carefully applied in a tough environment for a short time.

### 3.2. Control Test

**Figure 11:**
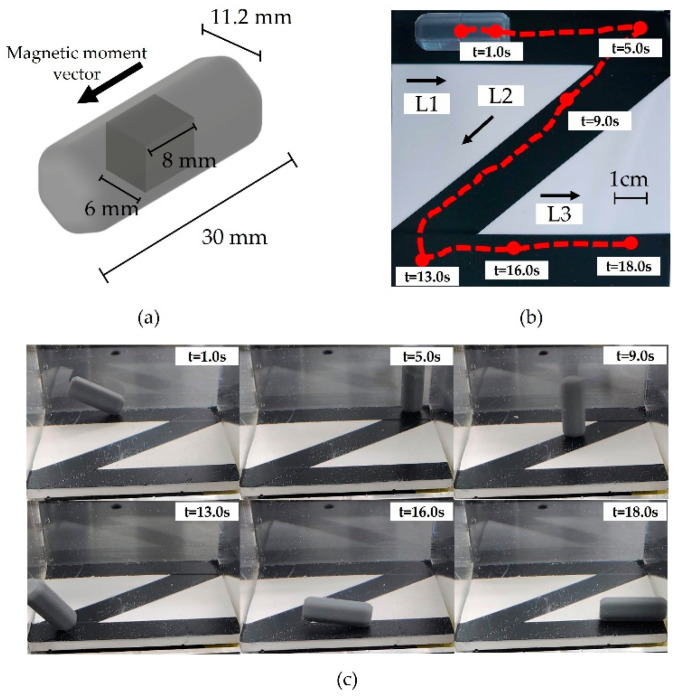
(**a**) Prototyped magnetic capsule used in experiment. (**b**) Z-path of the experiment and measured trajectory of capsule marked in red solid line. (**c**) Experiment of trajectory tracking.

To verify the magnetic field generation capability in 3-D space, the C-Mag was integrated with the SCARA which generated a rolling motion of the capsule according to the Z-path. Figure 11b shows the trajectory of the experiment. The experiment was conducted using a hexagonal capsule, shown in Figure 11a, because a round capsule may generate unstable motion. The experiment was conducted in a liquid with a viscosity of $8.0\times 10^{-4}$ Pa‧s, which is lower than that of the viscosity of the digestive system. During the experiment, C-Mag was translated and rotated along the L1, L2, and L3 directions by the SCARA while it generated a planar rotating magnetic field in the local plane. The measured trajectory of the capsule is shown in Figure 11b. Based on the results shown in Figure 11c and Supplementary Video S1, we confirmed that the system can effectively create a 3-D rolling motion of the capsule in a complex path.

## 4. Discussion

We proposed a novel MNS using two electromagnets and the SCARA. To maximize the magnetic field, various factors were considered such as the shape and angle of the core tip, payload of the SCARA, and capacity of the power supply unit. The constructed C-Mag was effectively rotated and translated by the SCARA to generate a 3-D rolling motion of the capsule. Although the capsule showed some ripple in the path following the experiment, the ripple would be decreased in the digestive system because the experimental environment was slippery and had a lower fluidic viscosity than the digestive system. Thus, we expect a more precise motion of the capsule in the digestive system.

However, the C-Mag has several remaining challenges. During the in vitro environment, the motion of the capsule was visually tracked, but additional tracking techniques should be considered for the human body [24,25,26,27]. Additionally, there are several uncertainties affected by the operating environment. The resistance can significantly change depending on the temperature of the coil, and the magnetic core has different hysteresis and eddy current loss depending on the operating frequency. These factors not only degrade the performance of the system, but also need to be censored for precise control. Especially in the case of temperature, cooling systems could be a possible option [28]. These issues are beyond the scope of this paper and will be addressed in future research.

## Data Availability

Not applicable.

## Supplementary Materials

The following supporting information can be downloaded at: https://www.mdpi.com/article/10.3390/mi14020269/s1, Video S1: path tracking experiment.
